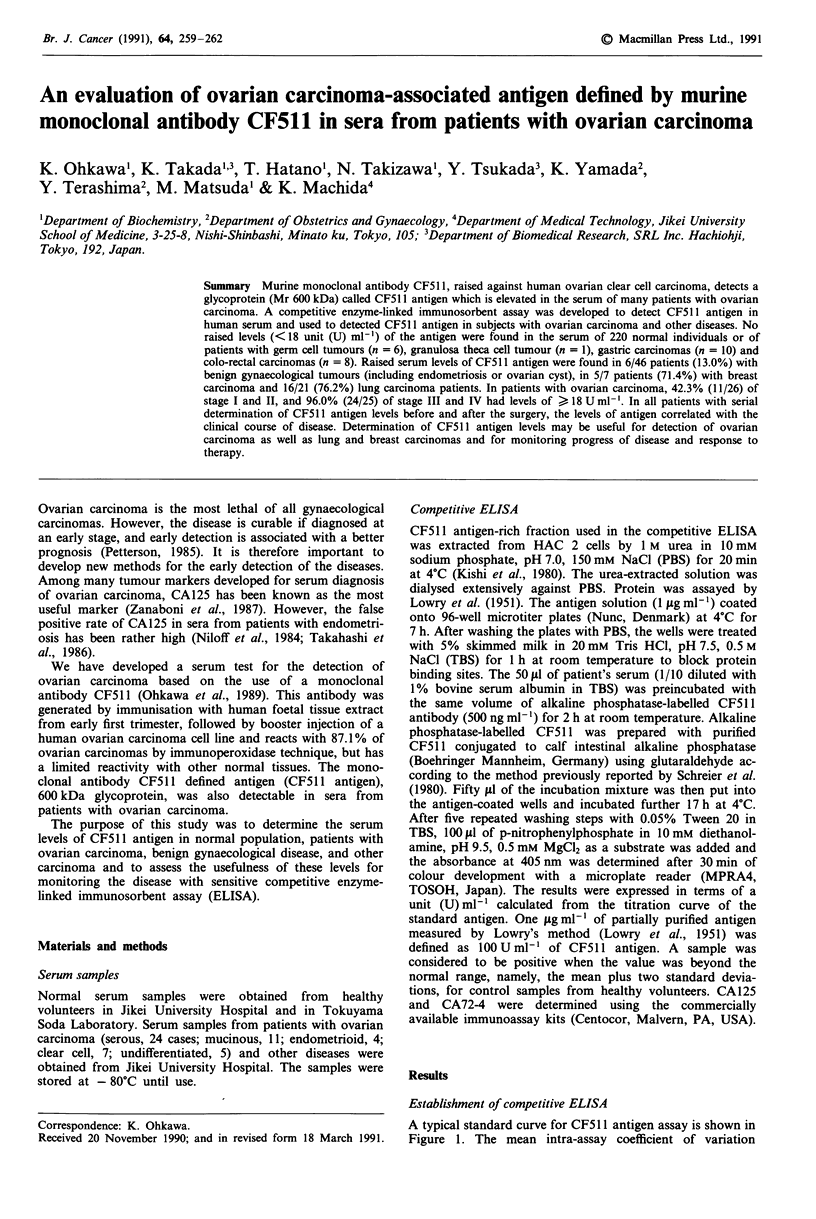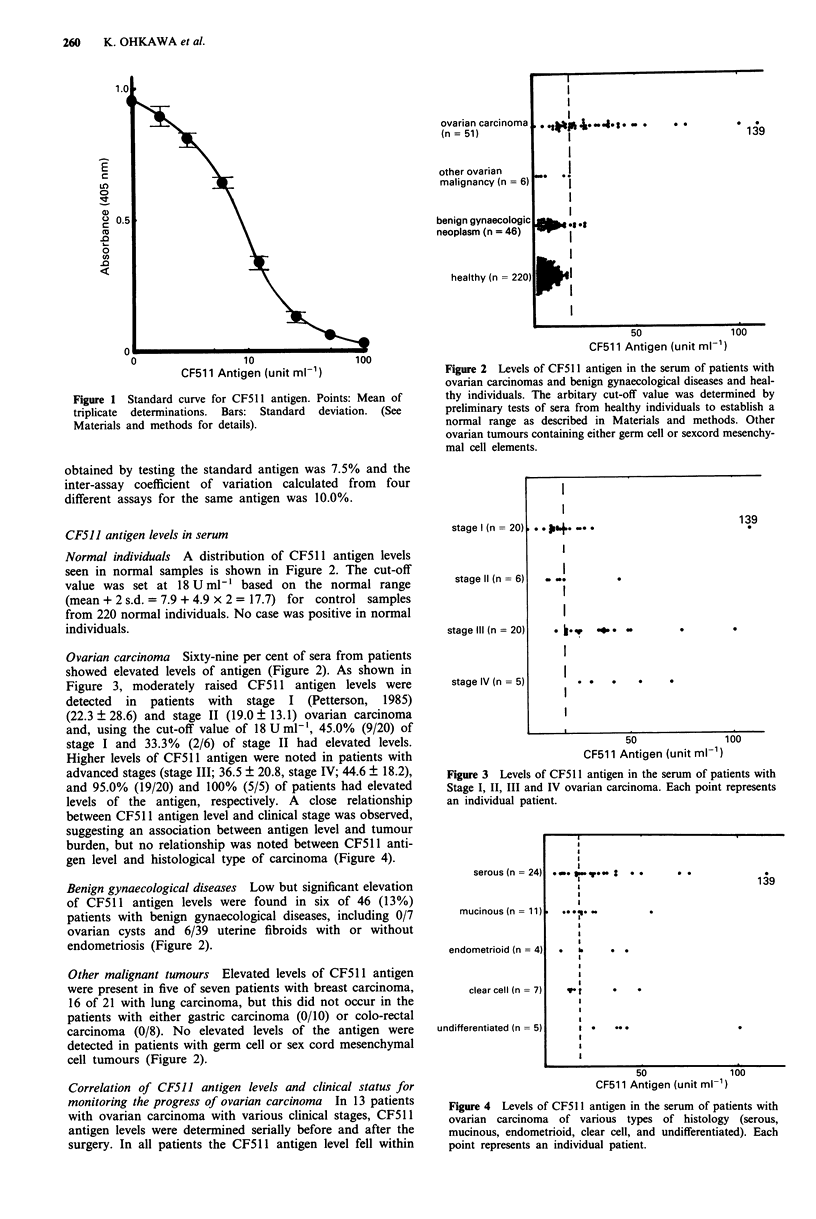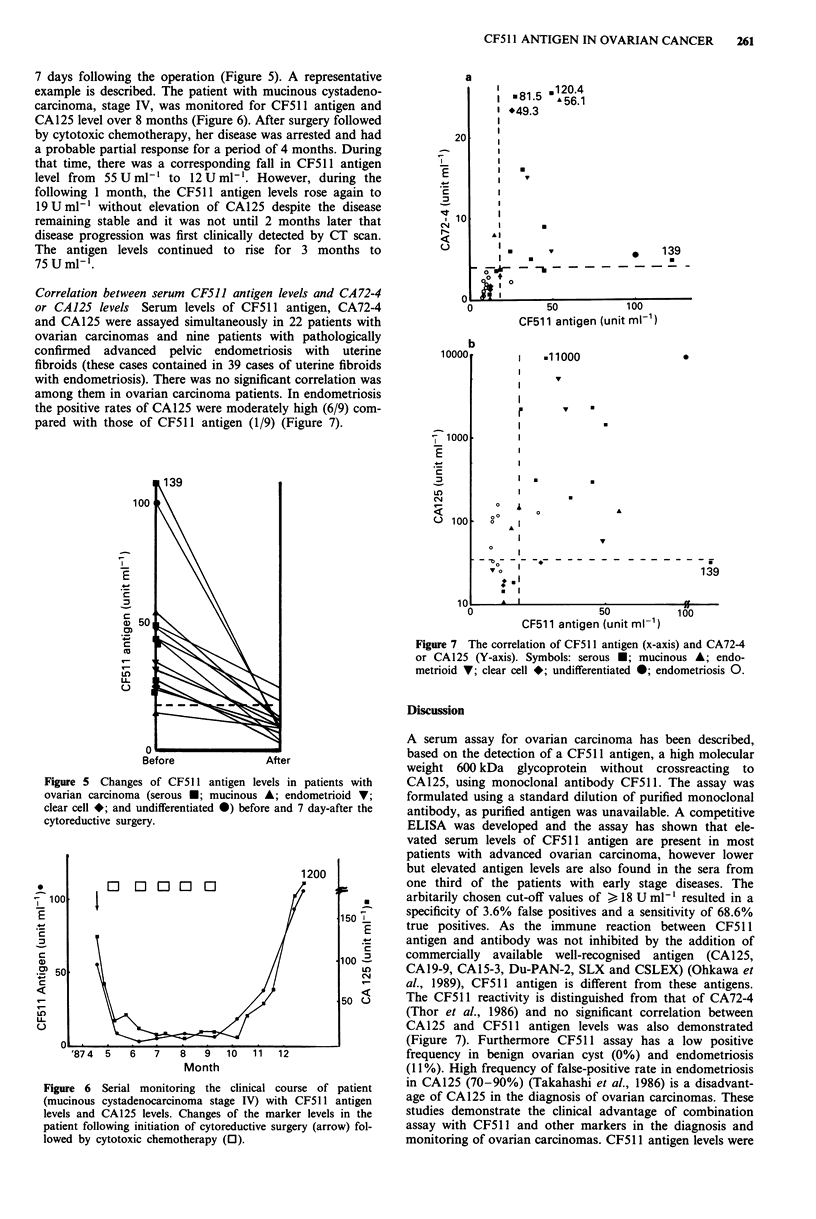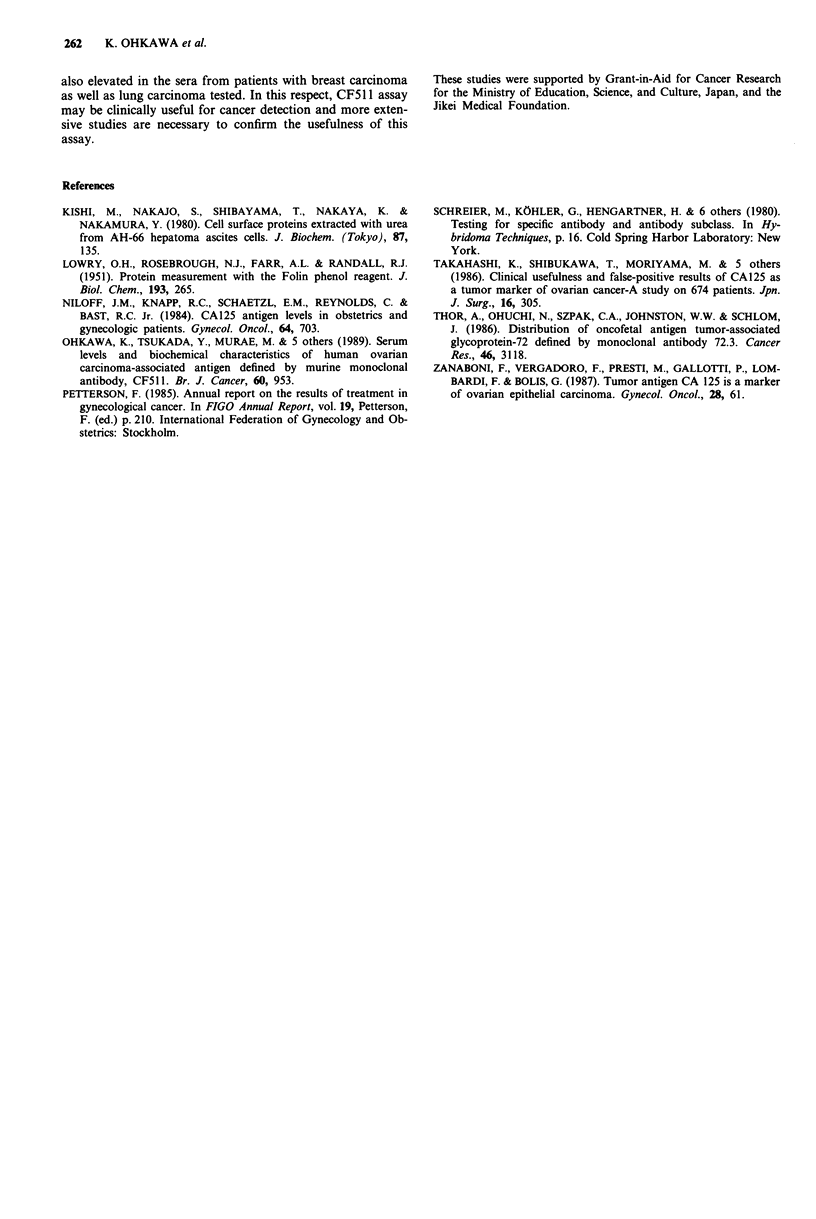# An evaluation of ovarian carcinoma-associated antigen defined by murine monoclonal antibody CF511 in sera from patients with ovarian carcinoma.

**DOI:** 10.1038/bjc.1991.288

**Published:** 1991-08

**Authors:** K. Ohkawa, K. Takada, T. Hatano, N. Takizawa, Y. Tsukada, K. Yamada, Y. Terashima, M. Matsuda, K. Machida

**Affiliations:** Department of Biochemistry, Jikei University School of Medicine, Tokyo, Japan.

## Abstract

Murine monoclonal antibody CF511, raised against human ovarian clear cell carcinoma, detects a glycoprotein (Mr 600 kDa) called CF511 antigen which is elevated in the serum of many patients with ovarian carcinoma. A competitive enzyme-linked immunosorbent assay was developed to detect CF511 antigen in human serum and used to detected CF511 antigen in subjects with ovarian carcinoma and other diseases. No raised levels (less than 18 unit (U) ml-1) of the antigen were found in the serum of 220 normal individuals or of patients with germ cell tumours (n = 6), granulosa theca cell tumour (n = 1), gastric carcinomas (n = 10) and colo-rectal carcinomas (n = 8). Raised serum levels of CF511 antigen were found in 6/46 patients (13.0%) with benign gynaecological tumours (including endometriosis or ovarian cyst), in 5/7 patients (71.4%) with breast carcinoma and 16/21 (76.2%) lung carcinoma patients. In patients with ovarian carcinoma, 42.3% (11/26) of stage I and II, and 96.0% (24/25) of stage III and IV had levels of greater than or equal to 18 U ml-1. In all patients with serial determination of CF511 antigen levels before and after the surgery, the levels of antigen correlated with the clinical course of disease. Determination of CF511 antigen levels may be useful for detection of ovarian carcinoma as well as lung and breast carcinomas and for monitoring progress of disease and response to therapy.


Br. J. Cancer (1991), 64, 259–262 © Macmillan Press Ltd., 1991

### An evaluation of ovarian carcinoma-associated antigen defined by murine monoclonal antibody CF511 in sera from patients with ovarian carcinoma

K. Ohkawa[1], K. Takada[1,3], T. Hatano[1], N. Takizawa[1], Y. Tsukada[3], K. Yamada[2], Y. Terashima[2], M. Matsuda[1] & K. Machida[4]

[1]*Department of Biochemistry,* [2]*Department of Obstetrics and Gynaecology,* [4]*Department of Medical Technology, Jikei University School of Medicine, 3-25-8, Nishi-Shinbashi, Minato ku, Tokyo, 105;* [3]*Department of Biomedical Research, SRL Inc. Hachiohji, Tokyo, 192, Japan.*

**Summary** Murine monoclonal antibody CF511, raised against human ovarian clear cell carcinoma, detects a glycoprotein (Mr 600 kDa) called CF511 antigen which is elevated in the serum of many patients with ovarian carcinoma. A competitive enzyme-linked immunosorbent assay was developed to detect CF511 antigen in human serum and used to detected CF511 antigen in subjects with ovarian carcinoma and other diseases. No raised levels (<18 unit (U) $ml^{-1}$) of the antigen were found in the serum of 220 normal individuals or of patients with germ cell tumours ($n = 6$), granulosa theca cell tumour ($n = 1$), gastric carcinomas ($n = 10$) and colo-rectal carcinomas ($n = 8$). Raised serum levels of CF511 antigen were found in 6/46 patients (13.0%) with benign gynaecological tumours (including endometriosis or ovarian cyst), in 5/7 patients (71.4%) with breast carcinoma and 16/21 (76.2%) lung carcinoma patients. In patients with ovarian carcinoma, 42.3% (11/26) of stage I and II, and 96.0% (24/25) of stage III and IV had levels of $\geqslant 18$ U $ml^{-1}$. In all patients with serial determination of CF511 antigen levels before and after the surgery, the levels of antigen correlated with the clinical course of disease. Determination of CF511 antigen levels may be useful for detection of ovarian carcinoma as well as lung and breast carcinomas and for monitoring progress of disease and response to therapy.

Ovarian carcinoma is the most lethal of all gynaecological carcinomas. However, the disease is curable if diagnosed at an early stage, and early detection is associated with a better prognosis (Petterson, 1985). It is therefore important to develop new methods for the early detection of the diseases. Among many tumour markers developed for serum diagnosis of ovarian carcinoma, CA125 has been known as the most useful marker (Zanaboni *et al.*, 1987). However, the false positive rate of CA125 in sera from patients with endometriosis has been rather high (Niloff *et al.*, 1984; Takahashi *et al.*, 1986).

We have developed a serum test for the detection of ovarian carcinoma based on the use of a monoclonal antibody CF511 (Ohkawa *et al.*, 1989). This antibody was generated by immunisation with human foetal tissue extract from early first trimester, followed by booster injection of a human ovarian carcinoma cell line and reacts with 87.1% of ovarian carcinomas by immunoperoxidase technique, but has a limited reactivity with other normal tissues. The monoclonal antibody CF511 defined antigen (CF511 antigen), 600 kDa glycoprotein, was also detectable in sera from patients with ovarian carcinoma.

The purpose of this study was to determine the serum levels of CF511 antigen in normal population, patients with ovarian carcinoma, benign gynaecological disease, and other carcinoma and to assess the usefulness of these levels for monitoring the disease with sensitive competitive enzyme-linked immunosorbent assay (ELISA).

### Materials and methods

### *Serum samples*

Normal serum samples were obtained from healthy volunteers in Jikei University Hospital and in Tokuyama Soda Laboratory. Serum samples from patients with ovarian carcinoma (serous, 24 cases; mucinous, 11; endometrioid, 4; clear cell, 7; undifferentiated, 5) and other diseases were obtained from Jikei University Hospital. The samples were stored at − 80°C until use.

Correspondence: K. Ohkawa.
Received 20 November 1990; and in revised form 18 March 1991.

### *Competitive ELISA*

CF511 antigen-rich fraction used in the competitive ELISA was extracted from HAC 2 cells by 1 M urea in 10 mM sodium phosphate, pH 7.0, 150 mM NaCl (PBS) for 20 min at 4°C (Kishi *et al.*, 1980). The urea-extracted solution was dialysed extensively against PBS. Protein was assayed by Lowry *et al.* (1951). The antigen solution (1 $\mu g\ ml^{-1}$) coated onto 96-well microtiter plates (Nunc, Denmark) at 4°C for 7 h. After washing the plates with PBS, the wells were treated with 5% skimmed milk in 20 mM Tris HCl, pH 7.5, 0.5 M NaCl (TBS) for 1 h at room temperature to block protein binding sites. The 50 µl of patient's serum (1/10 diluted with 1% bovine serum albumin in TBS) was preincubated with the same volume of alkaline phosphatase-labelled CF511 antibody (500 ng $ml^{-1}$) for 2 h at room temperature. Alkaline phosphatase-labelled CF511 was prepared with purified CF511 conjugated to calf intestinal alkaline phosphatase (Boehringer Mannheim, Germany) using glutaraldehyde according to the method previously reported by Schreier *et al.* (1980). Fifty µl of the incubation mixture was then put into the antigen-coated wells and incubated further 17 h at 4°C. After five repeated washing steps with 0.05% Tween 20 in TBS, 100 µl of p-nitrophenylphosphate in 10 mM diethanolamine, pH 9.5, 0.5 mM $MgCl_2$ as a substrate was added and the absorbance at 405 nm was determined after 30 min of colour development with a microplate reader (MPRA4, TOSOH, Japan). The results were expressed in terms of a unit (U) $ml^{-1}$ calculated from the titration curve of the standard antigen. One $\mu g\ ml^{-1}$ of partially purified antigen measured by Lowry's method (Lowry *et al.*, 1951) was defined as 100 U $ml^{-1}$ of CF511 antigen. A sample was considered to be positive when the value was beyond the normal range, namely, the mean plus two standard deviations, for control samples from healthy volunteers. CA125 and CA72-4 were determined using the commercially available immunoassay kits (Centocor, Malvern, PA, USA).

### Results

### *Establishment of competitive ELISA*

A typical standard curve for CF511 antigen assay is shown in Figure 1. The mean intra-assay coefficient of variation

**Figure 1** Standard curve for CF511 antigen. Points: Mean of triplicate determinations. Bars: Standard deviation. (See Materials and methods for details).

**Figure 2** Levels of CF511 antigen in the serum of patients with ovarian carcinomas and benign gynaecological diseases and healthy individuals. The arbitary cut-off value was determined by preliminary tests of sera from healthy individuals to establish a normal range as described in Materials and methods. Other ovarian tumours containing either germ cell or sexcord mesenchymal cell elements.

obtained by testing the standard antigen was 7.5% and the inter-assay coefficient of variation calculated from four different assays for the same antigen was 10.0%.

*CF511 antigen levels in serum*

*Normal individuals* A distribution of CF511 antigen levels seen in normal samples is shown in Figure 2. The cut-off value was set at 18 U $ml^{-1}$ based on the normal range (mean + 2 s.d. = 7.9 + 4.9 × 2 = 17.7) for control samples from 220 normal individuals. No case was positive in normal individuals.

*Ovarian carcinoma* Sixty-nine per cent of sera from patients showed elevated levels of antigen (Figure 2). As shown in Figure 3, moderately raised CF511 antigen levels were detected in patients with stage I (Petterson, 1985) (22.3 ± 28.6) and stage II (19.0 ± 13.1) ovarian carcinoma and, using the cut-off value of 18 U $ml^{-1}$, 45.0% (9/20) of stage I and 33.3% (2/6) of stage II had elevated levels. Higher levels of CF511 antigen were noted in patients with advanced stages (stage III; 36.5 ± 20.8, stage IV; 44.6 ± 18.2), and 95.0% (19/20) and 100% (5/5) of patients had elevated levels of the antigen, respectively. A close relationship between CF511 antigen level and clinical stage was observed, suggesting an association between antigen level and tumour burden, but no relationship was noted between CF511 antigen level and histological type of carcinoma (Figure 4).

*Benign gynaecological diseases* Low but significant elevation of CF511 antigen levels were found in six of 46 (13%) patients with benign gynaecological diseases, including 0/7 ovarian cysts and 6/39 uterine fibroids with or without endometriosis (Figure 2).

*Other malignant tumours* Elevated levels of CF511 antigen were present in five of seven patients with breast carcinoma, 16 of 21 with lung carcinoma, but this did not occur in the patients with either gastric carcinoma (0/10) or colo-rectal carcinoma (0/8). No elevated levels of the antigen were detected in patients with germ cell or sex cord mesenchymal cell tumours (Figure 2).

*Correlation of CF511 antigen levels and clinical status for monitoring the progress of ovarian carcinoma* In 13 patients with ovarian carcinoma with various clinical stages, CF511 antigen levels were determined serially before and after the surgery. In all patients the CF511 antigen level fell within

**Figure 3** Levels of CF511 antigen in the serum of patients with Stage I, II, III and IV ovarian carcinoma. Each point represents an individual patient.

**Figure 4** Levels of CF511 antigen in the serum of patients with ovarian carcinoma of various types of histology (serous, mucinous, endometrioid, clear cell, and undifferentiated). Each point represents an individual patient.

7 days following the operation (Figure 5). A representative example is described. The patient with mucinous cystadenocarcinoma, stage IV, was monitored for CF511 antigen and CA125 level over 8 months (Figure 6). After surgery followed by cytotoxic chemotherapy, her disease was arrested and had a probable partial response for a period of 4 months. During that time, there was a corresponding fall in CF511 antigen level from 55 U ml$^{-1}$ to 12 U ml$^{-1}$. However, during the following 1 month, the CF511 antigen levels rose again to 19 U ml$^{-1}$ without elevation of CA125 despite the disease remaining stable and it was not until 2 months later that disease progression was first clinically detected by CT scan. The antigen levels continued to rise for 3 months to 75 U ml$^{-1}$.

*Correlation between serum CF511 antigen levels and CA72-4 or CA125 levels* Serum levels of CF511 antigen, CA72-4 and CA125 were assayed simultaneously in 22 patients with ovarian carcinomas and nine patients with pathologically confirmed advanced pelvic endometriosis with uterine fibroids (these cases contained in 39 cases of uterine fibroids with endometriosis). There was no significant correlation was among them in ovarian carcinoma patients. In endometriosis the positive rates of CA125 were moderately high (6/9) compared with those of CF511 antigen (1/9) (Figure 7).

**Figure 5** Changes of CF511 antigen levels in patients with ovarian carcinoma (serous ■; mucinous ▲; endometrioid ▼; clear cell ◆; and undifferentiated ●) before and 7 day-after the cytoreductive surgery.

**Figure 6** Serial monitoring the clinical course of patient (mucinous cystadenocarcinoma stage IV) with CF511 antigen levels and CA125 levels. Changes of the marker levels in the patient following initiation of cytoreductive surgery (arrow) followed by cytotoxic chemotherapy (□).

**Figure 7** The correlation of CF511 antigen (x-axis) and CA72-4 or CA125 (Y-axis). Symbols: serous ■; mucinous ▲; endometrioid ▼; clear cell ◆; undifferentiated ●; endometriosis O.

### Discussion

A serum assay for ovarian carcinoma has been described, based on the detection of a CF511 antigen, a high molecular weight 600 kDa glycoprotein without crossreacting to CA125, using monoclonal antibody CF511. The assay was formulated using a standard dilution of purified monoclonal antibody, as purified antigen was unavailable. A competitive ELISA was developed and the assay has shown that elevated serum levels of CF511 antigen are present in most patients with advanced ovarian carcinoma, however lower but elevated antigen levels are also found in the sera from one third of the patients with early stage diseases. The arbitarily chosen cut-off values of $\geqslant 18$ U ml$^{-1}$ resulted in a specificity of 3.6% false positives and a sensitivity of 68.6% true positives. As the immune reaction between CF511 antigen and antibody was not inhibited by the addition of commercially available well-recognised antigen (CA125, CA19-9, CA15-3, Du-PAN-2, SLX and CSLEX) (Ohkawa *et al.*, 1989), CF511 antigen is different from these antigens. The CF511 reactivity is distinguished from that of CA72-4 (Thor *et al.*, 1986) and no significant correlation between CA125 and CF511 antigen levels was also demonstrated (Figure 7). Furthermore CF511 assay has a low positive frequency in benign ovarian cyst (0%) and endometriosis (11%). High frequency of false-positive rate in endometriosis in CA125 (70–90%) (Takahashi *et al.*, 1986) is a disadvantage of CA125 in the diagnosis of ovarian carcinomas. These studies demonstrate the clinical advantage of combination assay with CF511 and other markers in the diagnosis and monitoring of ovarian carcinomas. CF511 antigen levels were also elevated in the sera from patients with breast carcinoma as well as lung carcinoma tested. In this respect, CF511 assay may be clinically useful for cancer detection and more extensive studies are necessary to confirm the usefulness of this assay.

These studies were supported by Grant-in-Aid for Cancer Research for the Ministry of Education, Science, and Culture, Japan, and the Jikei Medical Foundation.

### References

KISHI, M., NAKAJO, S., SHIBAYAMA, T., NAKAYA, K. & NAKAMURA, Y. (1980). Cell surface proteins extracted with urea from AH-66 hepatoma ascites cells. *J. Biochem. (Tokyo)*, **87**, 135.

LOWRY, O.H., ROSEBROUGH, N.J., FARR, A.L. & RANDALL, R.J. (1951). Protein measurement with the Folin phenol reagent. *J. Biol. Chem.*, **193**, 265.

NILOFF, J.M., KNAPP, R.C., SCHAETZL, E.M., REYNOLDS, C. & BAST, R.C. Jr. (1984). CA125 antigen levels in obstetrics and gynecologic patients. *Gynecol. Oncol.*, **64**, 703.

OHKAWA, K., TSUKADA, Y., MURAE, M. & 5 others (1989). Serum levels and biochemical characteristics of human ovarian carcinoma-associated antigen defined by murine monoclonal antibody, CF511. *Br. J. Cancer*, **60**, 953.

PETTERSON, F. (1985). Annual report on the results of treatment in gynecological cancer. In *FIGO Annual Report*, vol. **19**, Petterson, F. (ed.) p. 210. International Federation of Gynecology and Obstetrics: Stockholm.

SCHREIER, M., KÖHLER, G., HENGARTNER, H. & 6 others (1980). Testing for specific antibody and antibody subclass. In *Hybridoma Techniques*, p. 16. Cold Spring Harbor Laboratory: New York.

TAKAHASHI, K., SHIBUKAWA, T., MORIYAMA, M. & 5 others (1986). Clinical usefulness and false-positive results of CA125 as a tumor marker of ovarian cancer-A study on 674 patients. *Jpn. J. Surg.*, **16**, 305.

THOR, A., OHUCHI, N., SZPAK, C.A., JOHNSTON, W.W. & SCHLOM, J. (1986). Distribution of oncofetal antigen tumor-associated glycoprotein-72 defined by monoclonal antibody 72.3. *Cancer Res.*, **46**, 3118.

ZANABONI, F., VERGADORO, F., PRESTI, M., GALLOTTI, P., LOMBARDI, F. & BOLIS, G. (1987). Tumor antigen CA 125 is a marker of ovarian epithelial carcinoma. *Gynecol. Oncol.*, **28**, 61.